# Supplementary material for: Expanding biochemical knowledge and illuminating metabolic dark matter with ATLASx
Source: Nat Commun. 2022 Mar 23;13:1560. doi: 10.1038/s41467-022-29238-z (PMC8943196; doi:10.1038/s41467-022-29238-z)
Supplement: Supplementary file 1 — Supplementary information [file 41467_2022_29238_MOESM1_ESM.pdf]

Supplementary Information

Expanding biochemical knowledge and illuminating metabolic dark matter with ATLASx

Homa MohammadiPeyhani[1,2†] & Jasmin Hafner[1,3,†], Anastasia Sveshnikova[1], Victor Viterbo[1], Vassily Hatzimanikatis[1*]

[1] Laboratory of Computational Systems Biotechnology, École Polytechnique Fédérale de Lausanne, EPFL, Lausanne, Switzerland

[2] Present address: Pharmaceutical Research and Early Development, Roche Glycart AG, 8952 Schlieren, Switzerland

[3] Present address: Department of Environmental Chemistry, EAWAG Swiss Federal Institute of Aquatic Science and Technology, Überlandstrasse 133, CH-8600 Dübendorf, Switzerland

[†]These authors contributed equally

*Corresponding author

Corresponding author email: Vassily.Hatzimanikatis@epfl.ch

Supplementary Discussion

Comparison of ATLASx with other pathway prediction tools

Reaction prediction and retrobiosynthesis tools

Diverse computational tools have emerged to bridge knowledge gaps in metabolism through cheminformatic predictions of potential metabolic reactions. Most of these tools have been developed for metabolic engineering applications, where the objective is to find biosynthetic routes that produce a desired target compound in a host organism [1–5]. Identification of these biosynthetic routes is accomplished by biochemically "walking back" from the target to precursor metabolites that are produced by, or fed to, the host organism. This procedure is called *retrobiosynthesis* and is implemented in a range of tools such as BNICE.ch [6,7], novoStoic [8], ReactPRED [9], and RetroPath [10,11]. While tools featuring reaction rules have the power to predict novel structures and biochemical reactions, their application is usually limited to a given research or engineering question. Furthermore, many tools require programing skills and extensive curation, filtration and screening of results. Since these tools use different standards, methods, and biochemical knowledge it is difficult to compare, reproduce, or transfer the results between different research groups.

Database approach based on known reactions

To provide easier access to reaction and pathway prediction for a broader range of researchers, various databases and online tools have been developed. The scope, size and application of these databases are different. MetaCyc [12] and KEGG [13] databases are golden standards to access experimentally observed (known) metabolic pathways. However, many metabolites and bioactive molecules (for example, drug molecules) remain out of scope of KEGG and MetaCyc since these molecules have been identified through mass spectrometry experiments, but their metabolism has not been elucidated yet.

Database approach based on known or novel reactions

To provide more comprehensive resources, the concept of enzymatic rules and reaction prediction is also employed by enviPath [14], a database for predicting biodegradation mechanisms, by MINEs [15], a database that predicts potential biological products for mass-spectrometry applications, and by ARBRE [16], a database centered around industrially important aromatic compounds. All these databases are available as user-friendly web tools. However, the scope of mechanisms integrated in these databases is focused on their specific field of application.

To systematically explore the metabolic dark matter arising from the elasticity of enzymatic catalysis, an unbiased approach is employed by Transform-MinER [17], novoPathFinder [18], and ATLAS of Biochemistry database [19,20] (developed in our group). These approaches generalize the concept of reaction rule and retrobiosynthesis and predict all theoretically possible novel reactions between metabolites reported in a single or in multiple databases. The generalized network of biochemistry around the KEGG database is published as ATLAS of Biochemistry and Transform-MinER. More recently, novoPathFinder applied this idea in larger scale by integrating the KEGG, ChEBI [21], and Rhea [22]databases. These works on unbiased enzymatic reaction prediction received a lot of attention from the scientific community. For instance, the generated repository of ATLAS allows the user to search for all known or predicted possible routes from any substrate compound to any product in the KEGG

database. However, one major drawback of these works is their limitation to a single or few compound sources, which excludes many drugs and plant natural products with undefined or putative biological functions. Predicting enzymatic reactions from biochemical compounds retrieved from other databases will help expand the scope of our predictions and enhance the application range and the predictive power of the database.

ATLASx

Following the previous publications but bringing the concept to the next level, in this article we introduce ATLASx, the first attempt to systematically map and fill the knowledge gaps in metabolism at the scale of the global biochemical knowledge. We decided to expand our database to all known, well-characterized and novel, predicted reactions between millions of known chemicals, biochemical and bioactive compounds. The ATLASx workflow unifies biochemical reactions and compounds from 14 different database sources (4 times bigger than any previous database) into one curated dataset called bioDB. bioDB holds 1.5 million unique biological or bioactive compounds and 56,000 unique biochemical reactions, which enable the prediction of a hypothetical biochemical space. By applying 490 bidirectional, generalized reaction rules from BNICE.ch onto biological and bioactive compounds within the database, we predicted around 1.6 million potential biotransformations between bioDB compounds. Another 3.6 million reactions were found to connect bioDB compounds with molecules only found in chemical databases, producing a total of 5.2 million predicted reactions. From these predictions, we characterized the connectivity and reactivity of biologically important molecules.
The development of ATLASx involves significant advances in database design, data analysis, and programming techniques. The ATLASx platform can be distinguished from the previous works by its comprehensive scope and wide range of applications. ATLASx can be readily used for the design of novel metabolic pathways, and for the exploration and expansion of biosynthesis pathways. Finally, and for the first time, ATLASx provides an estimation on the staggering number of unknowns in biochemistry, and can thus foster future research explorations into metabolic dark matter.

Supplementary Tables

Supplementary Table 1. Import and curation of compounds from different sources.

Name	Description	Reaction prediction	Pathway prediction	Online accessibility	# of integrated biochemical DBs	Scope
Tools, webservers						
BNICE.ch, RetroPath, NovoStoic, ReactPRED	Retrosynthesis algorithms that use generalized reaction rules to predict metabolic transformations	Yes	Yes	No	1 to 5	Limited to a given research or engineering question
Databases						
MetaCyc	Databases of experimentally elucidated metabolic pathways from literature	No	Yes	Yes	1	Limited to the known pathways in a single database
MINEs	A database that predicts potential biological products for mass-spectrometry applications	Yes	No	Yes	3	Focused on novel structures for mass-spectrometry applications
enviPath	A database and web server tool for predicting biodegradation mechanisms	Yes	Yes	Yes	Focused on xenobiotic chemicals	Limited to the microbial biotransformation of organic environmental contaminants
PathPred	Web based pathway search that uses substrate-product pairs in KEGG database for reaction prediction	Yes	Yes	Yes	1	Limited to KEGG database
novoPath-Finder	Web based pathway search that uses generalized reaction rules	Yes	Yes	Yes	3	Maximum pathway length 10
ATLAS of biochemistry, Transform-MinER	Databases of all possible biochemical reactions among KEGG compounds	Yes	Yes	Yes	1	Limited to KEGG database
ATLASx	A database of all known and predicted biochemical reactions in the unified space of biological and bioactive compounds	Yes	Yes	Yes	14	Global, no limit on the pathway length

Supplementary Table 2. Import and curation of compounds from different sources.

Database	Description	Collected	Imported	Unique in source database
Biological compounds				
MetaCyc	Manual/Cpds of sequenced organisms	15,819	14,828	12,524
Model SEED	Manual/KEGG and GSMs	33,995	20,665	17,132
KEGG Comp.	Manual/Cpds & biopolymers relevant to biology	18,625	17,397	15,064
Bioactive compounds				
KEGG Drug	Manual/approved drugs in Japan, USA, & Europe	11,140	7,766	4,514
Drugbank*	Approved drugs + discovery-phase drugs	8,350	6,279	3,850
ChEBI	Chemical Entities of Biological Interest	56,530	32,691	29,080
HMDB	Small cpds found in the human body	228,017	177,096	98,400
MetaNetX**	The metabolites in the GSMs + other databases	200,132	183,788	87,464
ChEMBL	Manual /bioactive/drug-like cpds	1,727,112	1,595,615	1,365,379
ATLASx biological and bioactive compounds (bioDB)				
Total (sum of single databases)		2,297,709	2,056,125	1,633,407
Total unique compounds				1,500,222

* Experimental drug ** Lipids excluded Cpd: Compound

Supplementary Table 3. Import and curation of reactions from different source databases.

**Database**	**Description**	**Collected**	**Imported**	**Unique in source database**
HMR	GSMs for human metabolic reactions	8,182	5,108	4,257
MetaCyc	Manual/reactions in pathways of sequenced orgs	16,052	15,438	12,093
KEGG	Manual/reactions in KEGG enzyme or KEGG pathway	10,829	10,685	10,338
MetaNetX	The reactions in the GSMs + other databases	42,182	40,767	25,871
Reactome	Manual/reactions in human	1,872	1,568	777
Rhea	Manual curation of biochemical rxns/cpds from ChEBI	20,770	19,325	11,753
Model SEED	Manual/KEGG and GSMs	44,031	44,010	25,807
BKMS	Rxns of BRENDA, KEGG, MetaCyc, and SABIO-RK	31,740	18,139	17,409
BiGG models	Manual/reactions from GSMs	28,299	16,581	8,354
Brenda	Large set of enzyme functional data	31,741	9,214	6,578
ATLASx reactions (bioDB)				
Total (sum of single databases)		235,698	180,835	123,237
Total unique reactions				56,087

GSM: Genome scale models Rxn: Reaction Cpd: Compound

Supplementary Table 4. Quality of reactions in different sources based on mass balance and EC annotation.

Database	# Total unique reactions	# EC annotated reactions	# Balanced* reactions	# Balanced & EC annotated reactions
KEGG	10,338	9,996	7,789	6,859
Brenda	6,578	5,982	5,855	5,281
Rhea	11,753	8,711	8,972	6,406
BiGG	8,354	3,657	3,972	1,755
Model SEED	25,807	8,662	16,041	6,300
MetaNetX	25,871	13,288	15,589	8,441
MetaCyc	12,093	8,495	8,458	6,282
HMR	4,257	3,153	2,969	2,001
Reactome	777	333	466	200
BKMS	17,409	14,589	10,962	9,252
Total in ATLASx	56,087	29,140	36,947	19,905

* removed isomerases, transports

Supplementary Table 5. Network statistics of bioDB, bioATLAS, and chemATLAS networks.

Property	bioDB	bioATLAS	chemATLAS
Weighted network			
Number of nodes	14,914	844,337	1,876,992
Number of edges (CAR > 0)	62,299	2,503,627	5,717,409
Unweighted network (only edges with CAR > 0.34)			
Number of nodes	14,084	617,942	1,854,423
Number of edges (CAR > 0.34)	25,624	982,343	2,778,445
Number of components (disjoint graphs)	623	68,912	151,390
Biggest component			
Number of nodes	12,434	361,405	1,264,423
Number of edges	24,575	774,584	2,297,335
Percent of total number of nodes	88.28%	58.49 %	68.19 %
Percent of total number of edges	95.84 %	78.85 %	82.68 %
Diameter [a]	32	27	46
Average path length [b]	7	9	12

[a] Length of longest shortest path between any two nodes, [b] Length of average shortest path length between any two nodes

Supplementary Table 6. Evolution of BNICE.ch reaction rules over time. Each third-level EC rule can be composed of one or several reaction mechanisms. Rules highlighted in orange have introduced a new third-level EC class to the collection of BNICE.ch reaction rules. Rules beginning with “NE_” represent non-enzymatic reactions that occur spontaneously under biological conditions.

2015		2018		2020		New in 2020	
**Third-level EC rule**	**# reaction mechanisms**	**Third-level EC rules**	**# reaction mechanisms**	**Third-level EC rules**	**# reaction mechanisms**	**Third-level EC rules**	**# reaction mechanisms**
1.1.1.-	16	1.1.1.-	16	1.1.1.-	17	1.1.1.-	1
1.10.3.-	1	1.1.3.-	1	1.1.3.-	1	1.10.3.-	1
1.11.1.-	4	1.10.3.-	1	1.10.3.-	2	1.11.1.-	1
1.13.11.-	12	1.11.1.-	4	1.11.1.-	5	1.13.11.-	5
1.13.12.-	2	1.13.11.-	12	1.13.11.-	17	1.13.12.-	1
1.14.11.-	1	1.13.12.-	2	1.13.12.-	3	1.14.12.-	1
1.14.12.-	6	1.14.11.-	2	1.14.11.-	2	1.14.13.-	5
1.14.13.-	14	1.14.12.-	6	1.14.12.-	7	1.14.14.-	4
1.14.14.-	1	1.14.13.-	14	1.14.13.-	19	1.14.18.-	2
1.14.15.-	2	1.14.14.-	1	1.14.14.-	5	1.14.19.-	5
1.14.16.-	1	1.14.15.-	2	1.14.15.-	2	1.14.99.-	2
1.14.18.-	1	1.14.16.-	1	1.14.16.-	1	1.21.98.-	3
1.14.19.-	1	1.14.18.-	1	1.14.18.-	3	1.23.1.-	1
1.14.99.-	1	1.14.19.-	2	1.14.19.-	7	1.3.1.-	2
1.17.1.-	1	1.14.21.-	1	1.14.21.-	1	1.3.3.-	1
1.17.3.-	1	1.14.99.-	1	1.14.99.-	3	1.5.1.-	1
1.17.4.-	1	1.17.1.-	1	1.17.1.-	1	1.5.3.-	1
1.17.99.-	1	1.17.3.-	1	1.17.3.-	1	1.7.1.-	1
1.2.1.-	10	1.17.4.-	1	1.17.4.-	1	1.97.1.-	1
1.2.3.-	2	1.17.7.-	1	1.17.7.-	1	2.1.1.-	7
1.2.4.-	1	1.17.99.-	1	1.17.99.-	1	2.3.1.-	3
1.2.5.-	1	1.2.1.-	11	1.2.1.-	11	2.4.1.-	1
1.2.99.-	1	1.2.3.-	2	1.2.3.-	2	2.4.2.-	2
1.3.1.-	7	1.2.4.-	2	1.2.4.-	2	2.4.99.-	1
1.3.3.-	2	1.2.5.-	1	1.2.5.-	1	2.5.1.-	6
1.4.1.-	3	1.2.99.-	1	1.2.99.-	1	2.6.99.-	1
1.4.3.-	2	1.23.1.-	1	1.21.98.-	3	2.7.1.-	1
1.4.4.-	1	1.3.1.-	7	1.23.1.-	1	2.7.8.-	1
1.5.1.-	9	1.3.3.-	2	1.3.1.-	9	3.1.1.-	1
1.5.3.-	1	1.4.1.-	3	1.3.3.-	3	3.1.8.-	2
1.5.99.-	1	1.4.3.-	2	1.4.1.-	3	3.3.2.-	2
1.6.5.-	2	1.4.4.-	1	1.4.3.-	2	3.5.3.-	1
1.7.1.-	9	1.5.1.-	9	1.4.4.-	1	4.1.1.-	2
1.7.3.-	1	1.5.3.-	3	1.5.1.-	10	4.1.3.-	2
1.8.1.-	7	1.5.99.-	1	1.5.3.-	4	4.1.99.-	1
1.8.4.-	1	1.6.5.-	2	1.5.99.-	1	4.2.1.-	3
1.97.1.-	3	1.7.1.-	9	1.6.5.-	2	4.2.99.-	2
2.1.1.-	9	1.7.3.-	1	1.7.1.-	10	4.3.3.-	1
2.1.2.-	1	1.8.1.-	7	1.7.3.-	1	4.4.1.-	1
2.1.3.-	1	1.8.4.-	1	1.8.1.-	7	4.5.1.-	1
2.2.1.-	5	1.97.1.-	3	1.8.4.-	1	4.99.1.-	1
2.3.1.-	10	2.1.1.-	11	1.97.1.-	4	5.3.3.-	1
2.3.3.-	2	2.1.2.-	2	2.1.1.-	18	6.3.2.-	1
2.4.1.-	5	2.1.3.-	1	2.1.2.-	2	NE_enol_ketone	1
2.4.2.-	5	2.2.1.-	6	2.1.3.-	1	NE_imine_form.	2
2.5.1.-	13	2.3.1.-	11	2.2.1.-	6	NE_N_OH_cycle	1
2.6.1.-	1	2.3.2.-	1	2.3.1.-	14	NE_ ring_closure	1
2.6.99.-	1	2.3.3.-	2	2.3.2.-	1	**New in 2020**	**89**
2.7.1.-	7	2.4.1.-	5	2.3.3.-	2		
2.7.3.-	1	2.4.2.-	7	2.4.1.-	6		
2.7.4.-	2	2.5.1.-	15	2.4.2.-	9		
2.7.7.-	1	2.6.1.-	2	2.4.99.-	1		
2.7.8.-	1	2.6.99.-	1	2.5.1.-	21		
2.8.2.-	1	2.7.1.-	7	2.6.1.-	2		
2.8.3.-	1	2.7.3.-	1	2.6.99.-	2		
3.1.1.-	3	2.7.4.-	2	2.7.1.-	8		
3.1.2.-	1	2.7.7.-	1	2.7.3.-	1		

3.1.3.-	1
3.1.4.-	1
3.1.6.-	1
3.13.1.-	1
3.2.1.-	2
3.2.2.-	1
3.3.1.-	1
3.3.2.-	1
3.5.1.-	3
3.5.3.-	3
3.5.4.-	3
3.5.5.-	1
3.5.99.-	1
3.6.1.-	1
3.7.1.-	5
3.8.1.-	3
4.1.1.-	14
4.1.2.-	11
4.1.3.-	3
4.2.1.-	18
4.2.3.-	5
4.2.99.-	1
4.3.1.-	5
4.3.2.-	2
4.4.1.-	5
4.5.1.-	1
4.6.1.-	3
4.99.1.-	1
5.1.2.-	1
5.3.1.-	4
5.3.2.-	2
5.3.3.-	7
5.3.99.-	4
5.4.2.-	4
5.4.3.-	2
5.4.4.-	5
5.4.99.-	2
5.5.1.-	9
5.99.1.-	1
6.2.1.-	5
6.3.1.-	1
6.3.2.-	3
6.3.4.-	5
6.3.5.-	3
6.4.1.-	2
**Total**	**360**

2.7.8.-	1
2.8.1.-	1
2.8.2.-	1
2.8.3.-	1
3.1.1.-	4
3.1.2.-	1
3.1.3.-	1
3.1.4.-	1
3.1.6.-	1
3.13.1.-	1
3.2.1.-	2
3.2.2.-	1
3.3.1.-	2
3.3.2.-	2
3.5.1.-	5
2.5.3.-	3
3.5.4.-	4
3.5.5.-	1
3.5.99.-	2
3.6.1.-	1
3.7.1.-	5
3.8.1.-	3
4.1.1.-	14
4.1.2.-	12
4.1.3.-	3
4.2.1.-	18
4.2.3.-	6
4.2.99.-	1
4.3.1.-	5
4.3.2.-	2
4.3.3.-	1
4.4.1.-	7
4.5.1.-	1
4.6.1.-	3
4.99.1.-	1
5.1.2.-	1
5.3.1.-	4
5.3.2.-	2
5.3.3.-	7
5.3.99.-	6
5.4.2.-	4
5.4.3.-	2
5.4.4.-	5
5.4.99.-	3
5.5.1.-	9
5.99.1.-	1
6.2.1.-	5
6.3.1.-	2
6.3.2.-	3
6.3.3.-	1
6.3.4.-	5
6.3.5.-	4
6.4.1.-	2
**Total**	**400**

2.7.4.-	2
2.7.7.-	1
2.7.8.-	2
2.8.1.-	1
2.8.2.-	1
2.8.3.-	1
2.8.4.-	1
3.1.1.-	5
3.1.2.-	1
3.1.3.-	1
3.1.4.-	1
3.1.6.-	1
3.1.8.-	2
3.13.1.-	1
3.2.1.-	2
3.2.2.-	1
3.3.1.-	2
3.3.2.-	4
3.5.1.-	5
3.5.3.-	4
3.5.4.-	4
3.5.5.-	1
3.5.99.-	2
3.6.1.-	1
3.7.1.-	5
3.8.1.-	3
4.1.1.-	16
4.1.2.-	12
4.1.3.-	5
4.1.99.-	1
4.2.1.-	21
4.2.3.-	6
4.2.99.-	3
4.3.1.-	5
4.3.2.-	2
4.3.3.-	2
4.4.1.-	8
4.5.1.-	2
4.6.1.-	3
4.99.1.-	2
5.1.2.-	1
5.3.1.-	4
5.3.2.-	2
5.3.3.-	8
5.3.99.-	6
5.4.2.-	4
5.4.3.-	2
5.4.4.-	5
5.4.99.-	3
5.5.1.-	9
5.99.1.-	1
6.2.1.-	5
6.3.1.-	2
6.3.2.-	4
6.3.3.-	1
6.3.4.-	5
6.3.5.-	4
6.4.1.-	2
NE_enol_ketone	1
NE_imine_form.	2
NE_N_OH_cycle	1
NE_ ring_closure	1
**Total**	**498**

Supplementary Table 7. Reconstruction of known bioDB reactions within ATLASx

Reconstruction category	Number of reactions
Reactions in bioDB	
Total reactions bioDB	56,087
Filtered bioDB reactions (only reactions with defined molecular structures of reactants are kept)	41,680
Reaction reconstruction: Exact coverage	
Reactions reconstructed with BNICE.ch rule	11,172
Reaction reconstruction: Alternative cofactor usage	
1-step reconstruction of main biotransformation(s) within ATLASx	14,193
2-step reconstruction: Main biotransformations reconstructed in max. of 2 reaction steps	2,625
3-step reconstruction: Main biotransformations reconstructed in max. of 3 reaction steps	1,175
4-step reconstruction: Main biotransformations reconstructed in max. of 4 reaction steps	603
Reaction reconstruction of bioDB	
Total number of reconstructed bioDB reactions	29,768
Percentage of reconstruction in filtered bioDB reactions	71.42

Supplementary Table 8. Processing and filtration of MetaCyc pathways: examples of culprits for pathway translation to linear

Reason	Ways to overcome	Potential drawback	Example pathways
Pathway represented as a sequence of reactions and not as sequence of intermediates	Translate to sequence of intermediates	Extracted sequence of intermediates does not represent the main flow of atoms	Potentially the case for all MetaCyc pathways
Precursor and target are not defined	Extract the precursor and target information from the MetaCyc properties "main reactants" and "main products"	"Main reactants" and "main products" are not provided for most of the pathways. "Main reactants" and "main products" can be several molecules for the same pathway	Potentially the case for all MetaCyc pathways
A molecule may act as a cofactor in some pathways, and as an intermediate in other pathways	Exclude common cofactors from the automatic pathway processing	Cofactor definition varies between the pathways, semi-manual work on cofactors definition	Potentially the case for all MetaCyc pathways
Branched pathway	Select one branch as the main pathway	Requires manual selection because of diversity of pathway architecture	PWY-361, PWY-7071,
Pathway consists of one reaction only	Exclude pathway	Reducing size of the dataset	PWY18C3-24, PWY-6316, PWY-7179, PWY-2841, PWY-2881, PWY-4181
Circular pathway	Exclude pathway	Reducing size of the dataset	ALKANEMONOX-PWY, PWY-7417, PWY66-398, PWY-7424, PWY-7124, PWY-4984
Molecular transport pathway	Exclude pathway	Reducing size of the dataset	PWY-6972
Pathway with intermediate without defined structure	Exclude pathway	Reducing size of the dataset	PWY4FS-8, PWY4FS-7, PWY-7694, PWY-5209
Pathway includes chelating steps	Exclude pathway	Reducing size of the dataset	PWY-7766
Pathway includes tRNA	Exclude pathway	Reducing size of the dataset	PWY0-1554
One of the intermediates is a protein	Exclude pathway	Reducing size of the dataset	PWY-5147, PWY-7282, PWY-6482, PWY-7546, PWY-7661
Within a pathway, a single compound acts both as a cofactor and as a pathway intermediate	Manually define pathway	Tedious	PWY-7054 (glutamine)
Low atom conservation between substrate and product in one step of the pathway	To recover the pathway in ATLASx, we have to lower the atom conservation threshold during the pathway reconstruction	Irrelevant connections appear	PWY-6965, PWY-82, PWY-6138, PWY3DJ-3547, PWY-6640
Includes spontaneous steps	Import reactions without enzyme into ATLASx	BNICE.ch rule assignment impossible	PWY-7895
Pathway focused on inorganic molecules transformations (non-carbon pathway)	Exclude pathway	Reducing size of the dataset	SO4ASSIM-PWY, PWY-6932
Pathway was deleted from MetaCyc	Exclude pathway	Reducing size of the dataset	PWY-4321, PWY-7548

Supplementary Figures

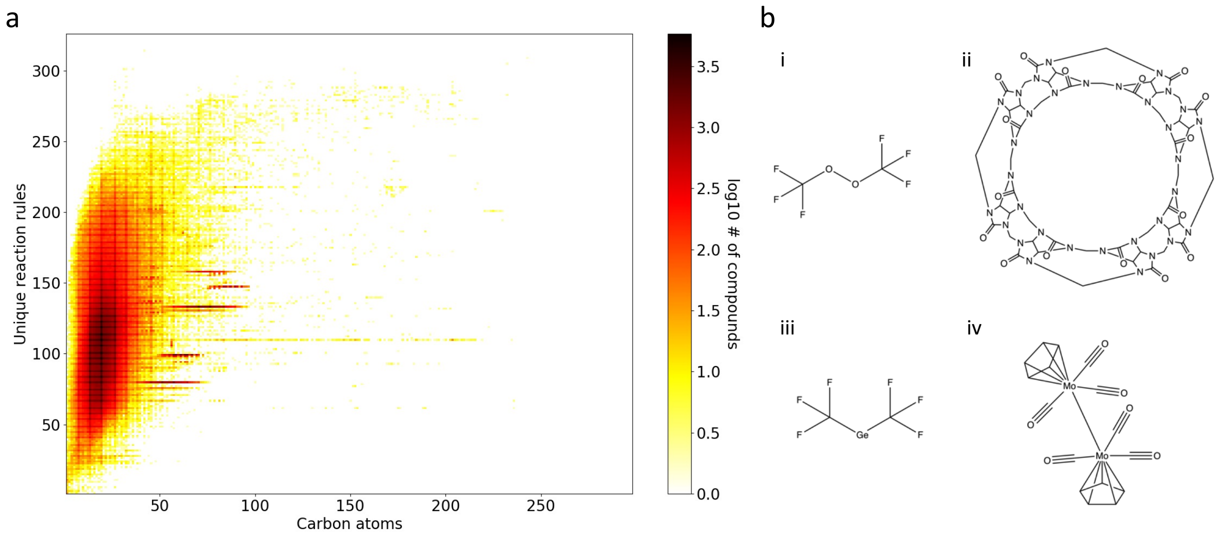

Supplementary Fig. 1. Reactive site analysis of bioATLAS compounds. a Heatmap showing the distribution of compounds as a function of their number of carbon atoms versus the number of reaction rules assigned to them. Darker colors indicate a higher number of compounds on a logarithmic scale. **b** Examples of four bioactive compounds for which BNICE.ch could not find any reactive site. i, Bis(trifluoromethyl)peroxide(BTP), ii, cucurbit[8]uril, iii, Bis(trifluoromethyl)germane, iv, bis[tricarbonyl(η5-cyclopentadienyl)molybdenum](Mo—Mo).

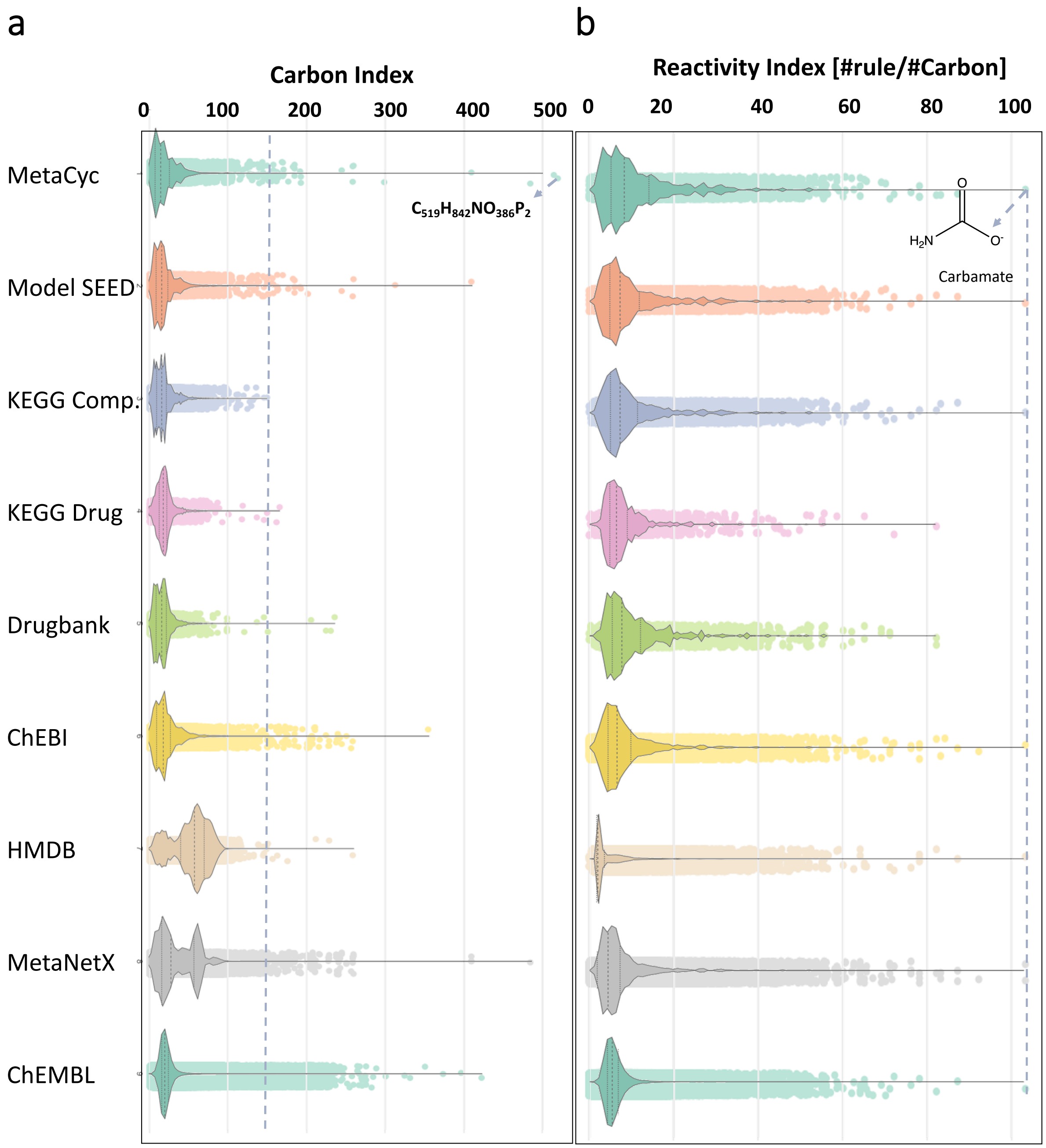

Supplementary Fig. 2. Biochemical reactivity of compounds compared across different databases. For each database, the distribution of compounds is represented as a violin plot. **a** The carbon index (i.e., number of carbon atoms inside the molecule) ranges from 0 to 511. The dashed line shows the maximum of carbon index in compounds of KEGG database, indicating broader distribution of molecules among bioactive molecules. **b** The reactivity index is calculated as the number of reaction rules assigned to a given compound, divided by the number of carbon atoms within the molecule. The reactivity index ranges from 0 to 104.

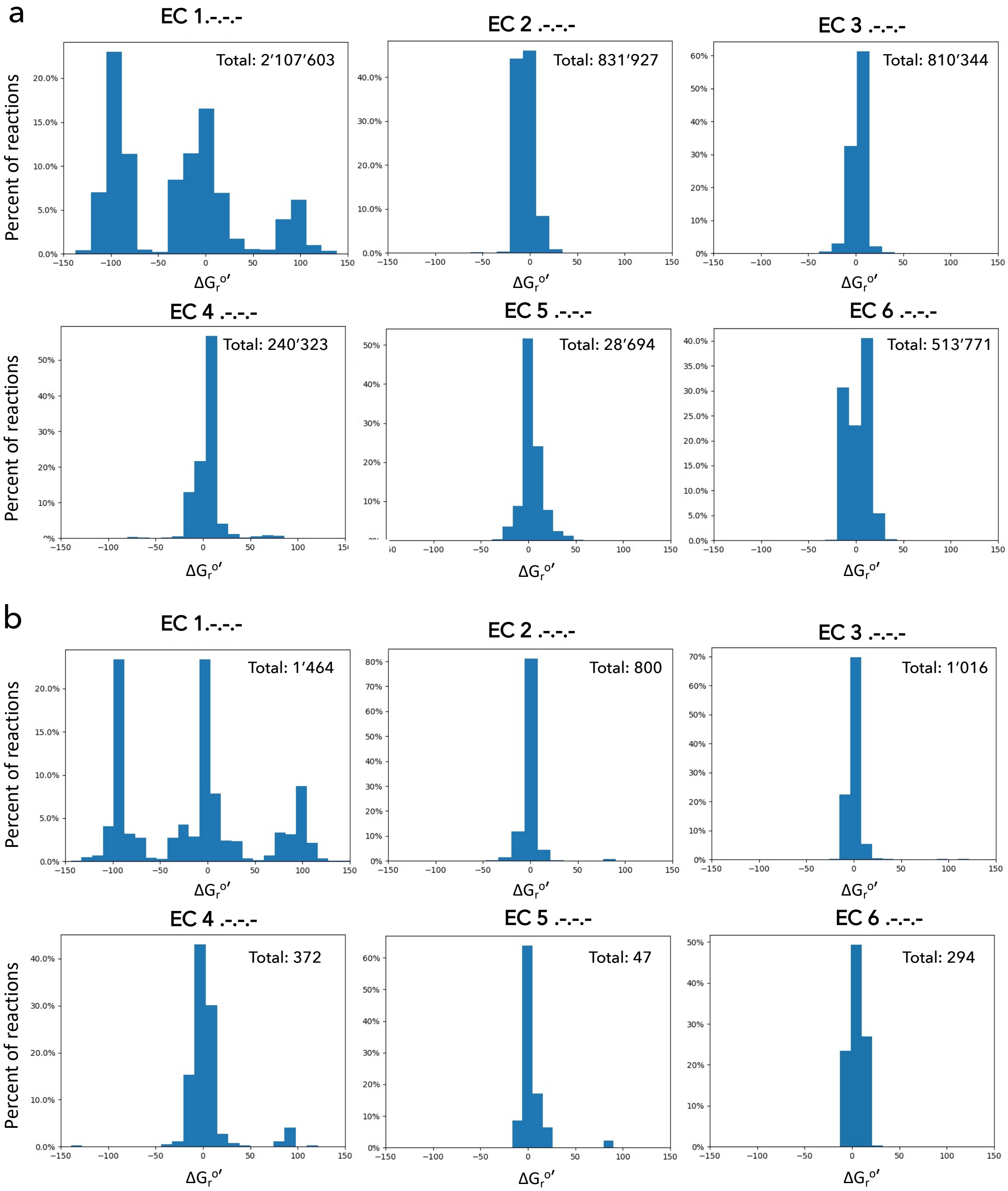

Supplementary Fig. 3. Histogram of the distribution of standard Gibbs energy of reaction for each 1st level EC class. In the upper right corner total number of reactions plotted indicated. **a** distribution of the Gibbs free energies calculated within chemATLAS space (total 4’231’154 (81%) reactions of chemATLAS have Gibbs free energy). **b** distribution of the Gibbs free energies calculated within bioDB space (total 3’809 reactions have Gibbs free energy estimation). Note that total number of the reactions that have a BNICE.ch reaction rule and energy estimation is not equal to the sum of totals per EC class as same reaction can have more than one first level EC class assigned. Besides this, not all reactions that have BNICE.ch rule assigned can have an energy estimation as energy cannot be estimated for reactions including generic compounds.

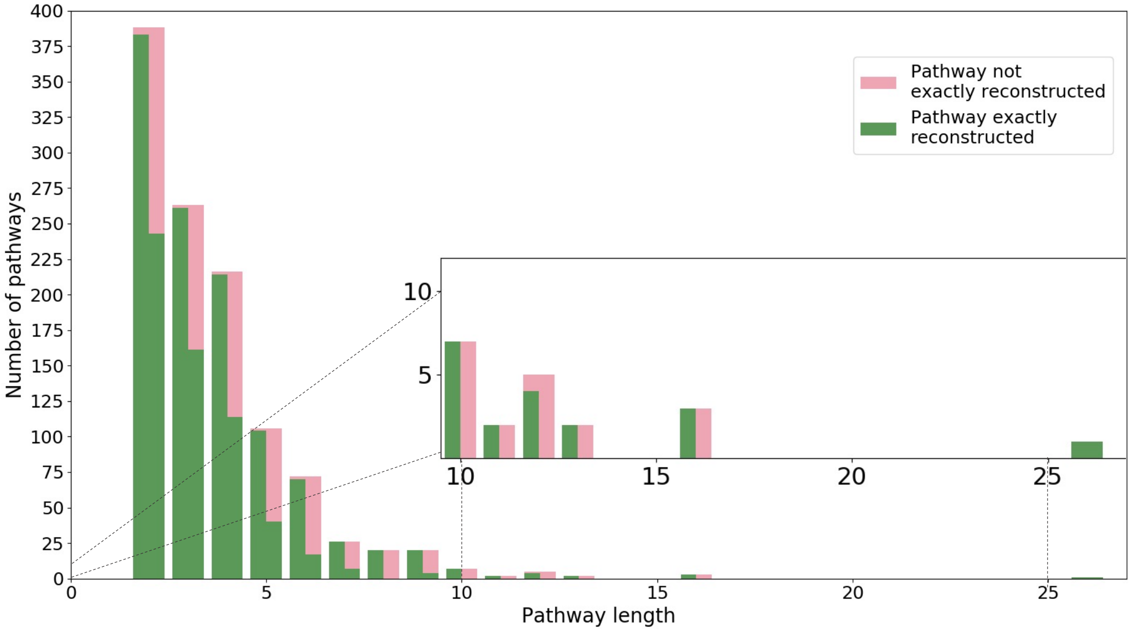

Supplementary Fig. 4. Pathway reconstruction for various length of the MetaCyc pathways. Coverage of the collected MetaCyc pathways dataset (1131 pathways) with all ATLASx network (left half of each column) and BNICE.ch mechanism annotated ATLASx (right half of each column) for different length of the pathway.

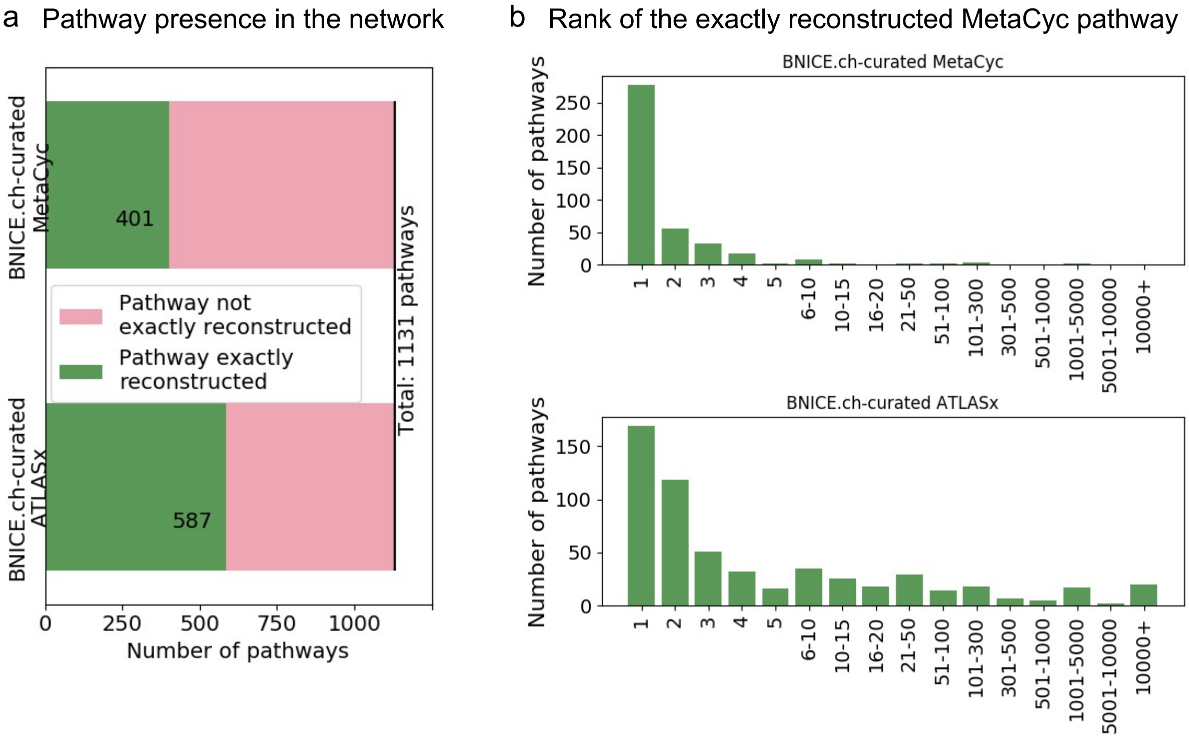

Supplementary Fig. 5. Pathway search comparison to dataset of pathways extracted from MetaCyc for only BNICE.ch-curated reactions. a Coverage of the collected MetaCyc pathways dataset (1131 pathways) with MetaCyc reactions in ATLAS and all ATLASx network annotated with BNICE.ch reaction rules. **b** Rank of the MetaCyc pathway according to the NICEpath pathway search algorithm.

References

1. Bachmann, B. O. Biosynthesis: Is it time to go retro? *Nat. Chem. Biol.* **6**, 390–393 (2010).
2. Hadadi, N. & Hatzimanikatis, V. Design of computational retrobiosynthesis tools for the design of de novo synthetic pathways. *Curr. Opin. Chem. Biol.* **28**, 99–104 (2015).
3. Wang, L., Ng, C. Y., Dash, S. & Maranas, C. D. Exploring the combinatorial space of complete pathways to chemicals. *Biochem. Soc. Trans.* **46**, 513–522 (2018).
4. Lin, G.-M. M., Warden-Rothman, R. & Voigt, C. A. Retrosynthetic design of metabolic pathways to chemicals not found in nature. *Curr. Opin. Syst. Biol.* **14**, 82–107 (2019).
5. Jeffryes, J. G., Seaver, S. M. D., Faria, J. P. & Henry, C. S. A pathway for every product? Tools to discover and design plant metabolism. *Plant Sci.* **273**, 61–70 (2018).
6. Hatzimanikatis, V. *et al.* Exploring the diversity of complex metabolic networks. *Bioinformatics* **21**, 1603–1609 (2005).
7. Tokic, M. *et al.* Discovery and evaluation of biosynthetic pathways for the production of five methyl ethyl ketone precursors. *ACS Synth. Biol.* **7**, 1858–1873 (2018).
8. Kumar, A., Wang, L., Ng, C. Y. & Maranas, C. D. Pathway design using de novo steps through uncharted biochemical spaces. *Nat. Commun.* **9**, 184 (2018).
9. Sivakumar, T. V., Giri, V., Park, J. H., Kim, T. Y. & Bhaduri, A. ReactPRED: a tool to predict and analyze biochemical reactions. *Bioinformatics* **32**, 3522–3524 (2016).
10. Delépine, B., Duigou, T., Carbonell, P. & Faulon, J.-L. RetroPath2.0: A retrosynthesis workflow for metabolic engineers. *Metab. Eng.* **45**, 158–170 (2018).
11. Koch, M., Duigou, T. & Faulon, J.-L. Reinforcement learning for bioretrosynthesis. *ACS Synth. Biol.* **9**, 157–168 (2020).
12. Caspi, R. *et al.* The MetaCyc database of metabolic pathways and enzymes. *Nucleic Acids Res.* **46**, D633–D639 (2018).
13. Kanehisa, M. & Goto, S. KEGG: kyoto encyclopedia of genes and genomes. *Nucleic Acids Res.* **28**, 27–30 (2000).
14. Wicker, J. *et al.* enviPath – The environmental contaminant biotransformation pathway resource. *Nucleic Acids Res.* **44**, D502–D508 (2016).

15. Jeffryes, J. G. *et al.* MINEs: open access databases of computationally predicted enzyme promiscuity products for untargeted metabolomics. *J. Cheminformatics* **7**, 44 (2015).
16. Sveshnikova, A., MohammadiPeyhani, H. & Hatzimanikatis, V. *ARBRE: Computational resource to predict pathways towards industrially important aromatic compounds*. 2021.12.06.471405 https://www.biorxiv.org/content/10.1101/2021.12.06.471405v1 (2021).
17. Tyzack, J. D., Ribeiro, A. J. M., Borkakoti, N. & Thornton, J. M. Exploring chemical biosynthetic design space with Transform-MinER. *ACS Synth. Biol.* **8**, 2494–2506 (2019).
18. Ding, S. *et al.* novoPathFinder: a webserver of designing novel-pathway with integrating GEM-model. *Nucleic Acids Res.* **48**, W477–W487 (2020).
19. Hadadi, N., Hafner, J., Shajkofci, A., Zisaki, A. & Hatzimanikatis, V. ATLAS of biochemistry: a repository of all possible biochemical reactions for synthetic biology and metabolic engineering studies. *ACS Synth. Biol.* **5**, 1155–1166 (2016).
20. Hafner, J., MohammadiPeyhani, H., Sveshnikova, A., Scheidegger, A. & Hatzimanikatis, V. Updated atlas of biochemistry with new metabolites and improved enzyme prediction power. *ACS Synth. Biol.* **9**, 1479–1482 (2020).
21. Hastings, J. *et al.* ChEBI in 2016: Improved services and an expanding collection of metabolites. *Nucleic Acids Res.* **44**, D1214 (2016).
22. Morgat, A. *et al.* Updates in Rhea--a manually curated resource of biochemical reactions. *Nucleic Acids Res.* **43**, D459-64 (2015).
