# Supplementary material for: Expanding biochemical knowledge and illuminating metabolic dark matter with ATLASx
Source: Nat Commun. 2022 Mar 23;13:1560. doi: 10.1038/s41467-022-29238-z (PMC8943196; doi:10.1038/s41467-022-29238-z)
Supplement: Supplementary file 2 — Reporting Summary [file 41467_2022_29238_MOESM2_ESM.pdf]

nature portfolio

Corresponding author(s): Vassily Hatzimanikatis

Last updated by author(s): Jan 31, 2022

Reporting Summary

Nature Portfolio wishes to improve the reproducibility of the work that we publish. This form provides structure for consistency and transparency in reporting. For further information on Nature Portfolio policies, see our Editorial Policies and the Editorial Policy Checklist.

Statistics

For all statistical analyses, confirm that the following items are present in the figure legend, table legend, main text, or Methods section.

n/a	Confirmed	
☒	☐	The exact sample size (*n*) for each experimental group/condition, given as a discrete number and unit of measurement
☒	☐	A statement on whether measurements were taken from distinct samples or whether the same sample was measured repeatedly
☒	☐	The statistical test(s) used AND whether they are one- or two-sided<br>*Only common tests should be described solely by name; describe more complex techniques in the Methods section.*
☒	☐	A description of all covariates tested
☒	☐	A description of any assumptions or corrections, such as tests of normality and adjustment for multiple comparisons
☒	☐	A full description of the statistical parameters including central tendency (e.g. means) or other basic estimates (e.g. regression coefficient) AND variation (e.g. standard deviation) or associated estimates of uncertainty (e.g. confidence intervals)
☒	☐	For null hypothesis testing, the test statistic (e.g. *F*, *t*, *r*) with confidence intervals, effect sizes, degrees of freedom and *P* value noted<br>*Give P values as exact values whenever suitable.*
☒	☐	For Bayesian analysis, information on the choice of priors and Markov chain Monte Carlo settings
☒	☐	For hierarchical and complex designs, identification of the appropriate level for tests and full reporting of outcomes
☒	☐	Estimates of effect sizes (e.g. Cohen's *d*, Pearson's *r*), indicating how they were calculated

Our web collection on statistics for biologists contains articles on many of the points above.

Software and code

Policy information about availability of computer code

Data collection	The tools used to build, annotate and search ATLASx have been previously published as BNICE.ch (version 2020), BridgIT (version 2022) and NICEpath (version 2021, https://github.com/EPFL-LCSB/nicepath), respectively. BNICE.ch and BridgIT use the OpenBabel library (version 2.4.0) for structural format conversion. NICEpath uses the NetworkX library (version 2.5) to represent and search biochemical networks.
Data analysis	The code to reproduce the presented analyses and figures is available at https://github.com/EPFL-LCSB/ATLASxAnalyses (DOI: 10.5281/zenodo.5925282). The python libraries NetworkX (version 2.5) and the SNAP (version 5.0.0) were used for the network analysis.

For manuscripts utilizing custom algorithms or software that are central to the research but not yet described in published literature, software must be made available to editors and reviewers. We strongly encourage code deposition in a community repository (e.g. GitHub). See the Nature Portfolio guidelines for submitting code & software for further information.

Data

Policy information about availability of data

All manuscripts must include a data availability statement. This statement should provide the following information, where applicable:

- Accession codes, unique identifiers, or web links for publicly available datasets
- A description of any restrictions on data availability
- For clinical datasets or third party data, please ensure that the statement adheres to our policy

Data supporting the findings in this work are available within the paper and its Supplementary Information files. Additional source data that are necessary to reproduce the analyses and figures presented in this paper are available on the publicly available git repository (https://github.com/EPFL-LCSB/ATLASxAnalyses). The data stored within the ATLASx database are available from the authors upon reasonable request. The following previously published datasets were used in this work: PubChem compound data (released in 2020, pubchem.ncbi.nlm.nih.gov), KEGG database (released in 2018, www.genome.jp/kegg/), ChEMBL database

(released in 2020, www.ebi.ac.uk/chembl/), MetaCyc (released in 2020, metacyc.org/), Model SEED (released in 2020, modelseed.org/), Drugbank (version5.1.6, go.drugbank.com/), ChEBI (released in 2020, www.ebi.ac.uk/chebi/), HMDB (Release 4.0, hmdb.ca/), MetaNetX (version 4.1, www.metanetx.org/), HMR (version 1.6, metabolicatlas.org/), Reactome (released in 2020, reactome.org/), Rhea (release 115, www.rhea-db.org/), BKMS (released in 2019, bkms.brenda-enzymes.org/), BiGG models (version 1.6, bigg.ucsd.edu/), and Brenda (released in 2019, www.brenda-enzymes.org/).

Field-specific reporting

Please select the one below that is the best fit for your research. If you are not sure, read the appropriate sections before making your selection.

- [x] Life sciences
- [ ] Behavioural & social sciences
- [ ] Ecological, evolutionary & environmental sciences

For a reference copy of the document with all sections, see nature.com/documents/nr-reporting-summary-flat.pdf

Life sciences study design

All studies must disclose on these points even when the disclosure is negative.

Sample size	*Describe how sample size was determined, detailing any statistical methods used to predetermine sample size OR if no sample-size calculation was performed, describe how sample sizes were chosen and provide a rationale for why these sample sizes are sufficient.*
Data exclusions	*Describe any data exclusions. If no data were excluded from the analyses, state so OR if data were excluded, describe the exclusions and the rationale behind them, indicating whether exclusion criteria were pre-established.*
Replication	*Describe the measures taken to verify the reproducibility of the experimental findings. If all attempts at replication were successful, confirm this OR if there are any findings that were not replicated or cannot be reproduced, note this and describe why.*
Randomization	*Describe how samples/organisms/participants were allocated into experimental groups. If allocation was not random, describe how covariates were controlled OR if this is not relevant to your study, explain why.*
Blinding	*Describe whether the investigators were blinded to group allocation during data collection and/or analysis. If blinding was not possible, describe why OR explain why blinding was not relevant to your study.*

Reporting for specific materials, systems and methods

We require information from authors about some types of materials, experimental systems and methods used in many studies. Here, indicate whether each material, system or method listed is relevant to your study. If you are not sure if a list item applies to your research, read the appropriate section before selecting a response.

Materials & experimental systems

n/a	Involved in the study	
[x]	[ ]	Antibodies
[x]	[ ]	Eukaryotic cell lines
[x]	[ ]	Palaeontology and archaeology
[x]	[ ]	Animals and other organisms
[x]	[ ]	Human research participants
[x]	[ ]	Clinical data
[x]	[ ]	Dual use research of concern

Methods

n/a	Involved in the study	
[x]	[ ]	ChIP-seq
[x]	[ ]	Flow cytometry
[x]	[ ]	MRI-based neuroimaging
